# Variations in eco-enzymatic stoichiometric and microbial characteristics in paddy soil as affected by long-term integrated organic-inorganic fertilization

**DOI:** 10.1371/journal.pone.0189908

**Published:** 2017-12-18

**Authors:** Sen Lin, Shaoxian Wang, Yuanli Si, Wenhao Yang, Shaowei Zhu, Wuzhong Ni

**Affiliations:** 1 College of Environmental and Resource Sciences, Zhejiang University, Key Laboratory of Agricultural Resource and Environment of Zhejiang Province, Hangzhou, P. R. China; 2 Jiangxi Academy of Agricultural Sciences, Nanchang, P. R. China; 3 College of Resource and Environmental Sciences, Fujian Agriculture and Forestry University, Fuzhou, P. R. China; RMIT University, AUSTRALIA

## Abstract

To investigate the effects of different nutrient management regimes on the soil chemical, eco-enzymatic stoichiometric and microbial characteristics, soil samples were collected from a 30-year, long-term field experiment with six plots growing rice. The results showed that as integrated fertilization increased, so did the concentrations of soil total or available nutrients and microbial biomass carbon (MBC). Our results also found enhanced soil basal respiration and cumulative carbon mineralization compared to chemical fertilization alone at the same nutrient doses. The activities of soil protease (Pro), β-glucosidase (βG), N-acetyl-glucosaminidase (NAG) and acid phosphatase (AP) from the integrated fertilization treatments were significantly higher than those of the treatments without organic manure, so did the activities of soil leucyl aminopeptidase (LAP) and urease (Ure) from the treatment with organic manure in addition to farmer practise fertilization (NPKM2). The stoichiometric ratios, expressed as lnβG/ln(NAG+LAP)/lnPro/lnUre/lnAP, ranged from 1:0.94:1.04:0.67:1.01 to 1:0.98:1.10:0.78:1.25, indicating that the acquisition of C, N and P changed consistently and synchronously under different nutrient management strategies. Integrated fertilization was more beneficial to the acquisition and utilization of soil organic carbon compared to low-molecular-weight organic nitrogen. We concluded that protease and urease should be considered in eco-enzymatic stoichiometric assessments for the hydrolysis of proteins, amino acids, carbohydrates and phosphomonoesters in soil, and integrated fertilization with chemical fertilizers and organic manure should be recommended as a preferable nutrient management system for intensive rice cultivation.

## Introduction

Nutrients introduced into farmland ecosystems come from mainly chemical fertilizers and organic manure. Routine applications of chemical fertilizers and organic manure are an essential component of soil management in arable crop production systems [1]. Chemical fertilizers are widely used to improve soil fertility and crop yield, significantly affecting soil biochemical and biological properties [2]. The total mass of chemical fertilizer applications was 59.96 million tons (pure nutrients) in 2014 in China [3], and the average amount applied per unit area is much higher than the world average. This excessive use of chemical fertilizer results in soil degradation, nutrients losses and low utilization rates of fertilizers and causes adverse impacts on the soil’s ecological functions and biochemical characteristics [4]. Moreover, excessive chemical fertilizers decrease soil enzymatic activities [5] and the concentration of soil microbial biomass carbon [6]. There was a greater increase in these parameters when organic manure was applied along with chemical fertilizers [1].

Organic fertilizer nutrient resources are very rich in China as large amount of livestock and poultry manure is generated each year. Based on statistics about livestock production in China, nutrient amounts in manure were estimated to be: N, 16.42 million tons; P_2_O_5_, 12.18 million tons; and K_2_O, 10.36 million tons in 2010, equivalent to 69.8%, 151.1% and 176.8% of the total chemical fertilizer applications this year, respectively [3,7]. Reducing chemical fertilizer applications and reasonably combining its use with organic fertilizers would be environmental friendly and preserve soil ecological functions, which are considered to be important factors in creating intensive and sustainable agriculture. Integrated fertilization with both chemical fertilizers and organic manure is the main strategy for nutrient cycling in agroecosystems and for developing sustainable agriculture, and has become a typical fertilization system in China.

Many reports have proposed that integrated fertilization with both organic manure and chemical fertilizer could improve soils’ physical and chemical properties [5,8–10]. The application of organic manure and organic-inorganic fertilizers can maintain and improve soil fertility, significantly increasing soil organic matter (SOM) and the available nutrient concentration in different crop systems [11–14]. The activities of β-glucosidase (βG) and acid phosphatase (AP) were higher under integrated organic and chemical nutrients management systems than under chemical fertilizers alone or no fertilizer use, while using chemical fertilizers only decreased the soil microbial mass carbon (MBC) and the soil basal respiration (SBR) level [15,16]. Soil enzymes mediate microbial nutrient acquisition from organic matter and are typically more sensitive to changes in soil management practices and environmental conditions than SOM. These activities are commonly used as indicators of microbial nutrient demand and soil quality changes [15,17–22]. Ecological stoichiometry theory is based on stoichiometric theory and the metabolic theory of ecology. This discipline uses elemental ratios and the concept of stoichiometric invariance to predict nutrient retention and microbial biomass production on subcellular to ecosystem scales, creating a unifying theory of ecology [23–25]. It is expressed as the C:N:P stoichiometric ratio, addressing the mass balance of multiple key elements in ecological systems, and has been successfully applied to topics ranging from population dynamics to biogeochemical cycling [22,26]. Both ecological stoichiometry theory and the complementary metabolic theory of ecology have developed over the course of a century [24]. However, their evaluations are complicated because ecological systems are controlled by the non-equilibrium flows of both materials (nutrients) and energy (carbon) and it is difficult to know which is predominant in a specific context [24]. The relationship between eco-enzymatic stoichiometric characteristics and the soil biochemistry of organic matter mineralization and nutrient cycling is still unclear.

The mineralization of organic matter in soils mainly involves the release of organic nitrogen and phosphorus. Soil nitrogen-containing organic matter releases proteins during the initial degradation process. Most of the proteins are gradually decomposed and mineralized under the action of microorganisms and soil protease (Pro). The mineralization of soil proteins mainly includes the amination and ammonification processes. The improvement of soil protease activity enhances the mineralization of soil nitrogen-containing organic matter and releases more amino acids and amides. This undoubtedly provides abundant substrates for urease (Ure), N-Acetyl-glucosaminidase (NAG) and leucyl aminopeptidase (LAP) in the ammonification stage. Organic phosphorus in most mineral soils occurs as a mixture of phosphate monoesters and phosphate diesters, with smaller amounts of phosphonates and organic polyphosphates [27]. Phosphatase mineralizes P from nucleic acids, phospholipids and other ester phosphates [28,29]. Ecological studies generally quantify only the activities of enzymes which catalyse the terminal reactions that produce assimilable products from the principal C, N and P sources [24]. The soil enzymes most widely assayed using fluorogenic substrates with high-throughput microplate technology, βG, NAG, LAP, and AP, hinge functional stoichiometries in relation to organic nutrient acquisition and are used as indicators of microbial nutrient demands [24,30]. Nutrient cycles and organic material transformations in the soil involve many classes of enzymes. Protease is a key enzyme involved in the N cycle [31,32]. The application of urea fertilizer in agricultural practices increases the amide nitrogen content in the soil. Moreover, organic nitrogen in humic substances mainly occurs in amide functional groups [33]. Urease has absolute specificity to amidohydrolases in the soil and plays an irreplaceable role in the N cycle. Therefore, it is necessary to take soil protease and urease into consideration when we study organic material mineralization and nutrient cycles. In addition, enzymatic activities and microbial biomass are closely related because the transformations of important organic elements occur through microorganisms [32]. However, studies of the correlation between the whole enzymatic reaction process of complex organic compounds transforming into simple organic and microbial biomass are relatively rare.

The objectives of the present study were to assess the impacts of long-term (30 years) integrated fertilization with organic manure and chemical fertilizers on the enzymatic stoichiometric and microbial characteristics and the relationships involved. This study further aimed to evaluate the effects of different fertilization management strategies on the sustainable use potential of soil ecosystems.

## Materials and methods

### Site description and sampling

This study was conducted in the Poyang Lake Plain, which is a major rice (*Oryza sativa* L.) production area in China. The site has a mid-subtropical and monsoon climate with an annual average temperature and precipitation of 17.6°C and 1718.4 mm, respectively. The field experiment with the rice-rice rotation began in April of 1983 in Shanggao County (28°02′N, 114°28′E), Jiangxi Province, China. The experiment with the rice-rice crop rotation included six treatments such as no fertilizer (CK), chemical nitrogen fertilizer (N), chemical nitrogen and phosphorus fertilizers (NP), complete chemical fertilizers (NPK), integrated fertilization with organic and chemical fertilizers at the same nutrient doses of NPK (NPKM1), and an amount of organic manure equal to NPKM1 in addition to the NPK treatment (NPKM2). Each treatment had three replicates for a total 18 plots in this experiment. The plot area was 30 m^2^. The nutrient doses of different treatments are shown in Table 1. The chemical fertilizers and organic manure applied were urea, calcium magnesium phosphate, potassium chloride and ordinary farm manure. The chemical-physical property of the organic manure was determined every year and the nutrient concentrations were different. Fertilizing amount of organic manure was calculated according to its nitrogen concentration to make sure the experiment was consistent with the same proportion of organic and inorganic nitrogen. In 2013, organic manure applied in early rice season contained 15.78% OC (organic carbon, dry basis), 1.07% N, 0.27% P_2_O_5_, and 0.73% K_2_O; organic manure applied in late rice season contained 12.20% OC, 0.80% N, 0.30% P_2_O_5_, and 0.67% K_2_O.

**Table 1:** Nutrient doses of different treatments.

Treatment	Nutrient dose (kg ha^-1^)								
	N			P_2_O_5_			K_2_O		
	Chemical	Organic	Total	Chemical	Organic	Total	Chemical	Organic	Total
Early Rice									
CK	0	0	0	0	0	0	0	0	0
N	120	0	120	0	0	0	0	0	0
NP	120	0	120	45	0	45	0	0	0
NPK	120	0	120	45	0	45	75	0	75
NPKM1	72	48	120	33	12	45	42	33	75
NPKM2	120	48	168	45	12	57	75	33	108
Late Rice									
CK	0	0	0	0	0	0	0	0	0
N	150	0	150	0	0	0	0	0	0
NP	150	0	150	45	0	45	0	0	0
NPK	150	0	150	45	0	45	75	0	75
NPKM1	126	24	150	36	9	45	55	20	75
NPKM2	150	24	174	45	9	54	75	20	95

Soil samples were collected at each plot at depths of 0–20 cm by using a tube-shaped stainless-steel sampler with a diameter of 2 cm after the harvest of late rice in November 2013 and were divided into two parts. Within each plot, soil samples were collected randomly according to S-style sampling method, then mixed the samples into one sample, and reduced by “coning and quartering”. Fresh soil samples were kept in the refrigerator at 4°C for analyses. Determinations of the enzymatic activities and microbial properties were completed within one week. The remainder of the soil samples was air-dried, ground and sieved for soil chemical properties analysis.

### Soil chemical properties

The soil pH value was measured with a handy-pH metre (PT-10, Sartorious, Germany) using a soil-to-water ratio of 1:2.5. The soil organic carbon (SOC), total nitrogen (TN), total phosphorus (TP), total potassium (TK), alkali-hydrolysable N (Ah-N), available phosphorus (Olsen-P) and exchangeable potassium (EK) were analysed using the conventional methods described by Lu [34].

### Soil enzyme activities

For the determination of soil urease (EC 3.5.1.5) activity, 0.5 mL of toluene, 20 mL of pH 6.7 citrate buffer and 10 mL of 10% urea were added to 5.0 g soil, after which the mixture was incubated at 37°C for 24 hours. The released ammonium was measured colourimetrically using the Indophenol Blue Method at 578 nm [35]. A control without urea was measured with each sample. The activity was expressed in units of nmol g^-1^ h^-1^.

Soil protease activity was assayed using the method as Cao et al. [36]. About 2 g of soil was weighed into a glass vial and 10 mL of 1% gelatin was prepared with pH 7.4 phosphate buffer and 0.5 mL of methylbenzene. The vials were incubated at 30°C for 24 hours. After incubation, all the contents were filtered and 5 mL was placed into a tube, and the remaining protease in the tube was precipitated with 0.5 mL of 0.05 mol L^-1^ sulfuric acid and 3 mL of 20% Na_2_SO_4_. The suspension was then filtered into a 50 mL volumetric flask and 1.0 mL of 2% ninhydrin was added. The volumetric flask was placed in a boiling water bath for 10 minutes. The released glycine was measured colourimetrically at 560 nm. Blanks without soil were also analysed. The activity was expressed in units of nmol g^-1^ h^-1^.

Soil enzyme activities, including βG (EC 3.2.1.21), NAG (EC 3.2.1.14), LAP (EC 3.4.11.1) and AP (EC 3.1.3.2), were measured using a microplate fluorimetric assay [23,37]. The substrates for these enzymes were MUB-linked or AMC-linked model substrates (4-MUB-β-_D_-glucoside, 4-MUB-N-acetyl-β-_D_-glucosaminide, _L_-Leucine-7-amido-4-methylcoumarin and 4-MUB-phosphate from the Sigma-Aldrich Corporation, USA) yielding the highly fluorescent cleavage products 4-methylumbelliferyl (MUB) or 7-amino-4-methylcoumarin (AMC) upon hydrolysis, respectively. Substrate solutions were prepared in 50 mmol L^-1^ sodium acetate buffer (pH 5.0). The activities were expressed in units of nmol g^-1^ h^-1^.

### Soil microbial biomass C

The soil microbial biomass carbon (MBC) was determined following the Fumigation Extraction method [38,39] using the following equation: MBC = EC/*K*_EC_, where EC is the difference between the organic C extracted from fumigated soils and the organic C extracted from non-fumigated soils and *K*_EC_ = 0.45.

### Soil basal respiration (SBR) and organic carbon mineralization (C_min_)

The alkali absorption method was performed for quantifying CO_2_ evolution. Specifically, moist soils (10 g dry weight equivalent) were adjusted to 50% of field capacity and spread out on the bottom of 250 mL glass jars in which an absorption bottle with 4 mL of NaOH (0.1 mol L^-1^) solution was suspended, then incubated at 25°C for 28 days. After incubation for 1, 3, 5, 7, 14, 21 or 28 days, 2 mL of 1.0 mol L^-1^ BaCl_2_ and 2 drops of phenolphthalein indicator were added to the bottles, then a titration was performed with 0.1 mol L^-1^ HCl. The jars without soil served as the controls. The difference in the consumed volume of HCl between the treatments and the controls in the titration was used to calculate the quantity of CO_2_ evolution from soil microbes, with 1 mL of 0.1 mol L^-1^ consumed NaOH being equivalent to 2.2 mg CO_2_ [40].

### Statistical analysis

Data processing was carried out using Microsoft Excel 2010, and the means and standard errors (SE) of the parameters were calculated. Analysis of Variance (ANOVA) was employed to determine the significance of means at *p <* 0.05 using Duncan’s Multiple Range Test in SPSS (Version 20.0), and Pearson correlation coefficients were calculated. GraphPad Prism (Version 6.0) was used to prepare figures. The acquisition ratios of lnβG/lnAP, lnβG/ln(NAG+LAP), and ln(NAG+LAP)/lnAP activities were calculated, referring to the acquisition of organic C relative to organic P and N and to the acquisition of organic N relative to organic P, respectively [24]. In the same way, lnβG/lnUre, lnβG/lnPro, lnPro/ln(NAG+LAP), lnPro/lnUre, lnUre/lnAP and lnPro/lnAP were also calculated.

## Results

### Soil nutrient concentrations and pH values

The soil nutrient concentrations and pH values of different treatments are presented in Table 2. The soil TN and Ah-N concentrations of the nitrogen supply treatments were significantly higher than those of CK. The soil TP and Olsen-P concentrations of the phosphorus supply treatments were significantly higher than those of the CK and N treatments. Under the NPKM1 treatment, the soil Ah-N, Olsen-P and SOC were significantly higher than those treatments without organic manure; this treatment did not significantly differ from the NPK treatments in soil TN, TK and EK concentrations. Under the NPKM2 treatment, the soil TN and Olsen-P concentrations were significantly higher than those of the other treatments; the soil Ah-N, TP, Olsen-P and SOC were significantly higher than those of treatments without organic manure (CK, N, NP, NPK). The soil pH value was approximately 6.0 and the differences between treatments did not reach significant levels.

**Table 2:** Soil nutrient concentrations and pH values after long-term different fertilization.

Treatments	Total nutrient (g kg^-1^)			Available nutrient (mg kg^-1^)			SOC (g kg^-1^)	pH
	TN	TP	TK	Ah-N	Olsen-P	EK		
CK	1.34d	0.43d	14.5bc	191.6c	10.1d	31.6c	14.6d	6.10a
N	1.58b	0.44d	14.6bc	237.3b	9.9d	30.9c	15.9c	6.02a
NP	1.55b	0.61bc	13.8c	235.1b	24.6c	29.8c	16.9b	6.03a
NPK	1.46c	0.58c	15.0ab	230.2b	21.6c	40.1b	16.6b	6.02a
NPKM1	1.50bc	0.63ab	15.3ab	260.9a	29.4b	42.8ab	19.0a	6.05a
NPKM2	1.68a	0.66a	15.7a	267.5a	35.1a	43.9a	19.6a	6.04a

Note: Different letters in the same column indicate significant differences among fertilizer treatments at the *p <* 0.05 level.

The molar ratios of SOC to TN (C/N), SOC to TP (C/P) and TN to TP (N/P) are shown in Table 3. The C/N ratio of treatment N was significantly lower than in the other treatments. The C/N ratios of treatments with organic manure (NPKM1, NPKM2) were significantly higher than those of treatments CK, N and NP. The C/P and N/P ratios of the phosphorus supply treatments (NP, NPK, NPKM1, NPKM2) were significantly lower than those with no phosphorus treatments (CK, N). The C/P and N/P ratios of the CK treatment were significantly lower than those of treatment N. The differences in C/P and N/P ratios among the phosphorus supply treatments were insignificant.

**Table 3:** Molar ratios of soil SOC, soil TN and TP after long-term different fertilization.

Treatments	C/N	C/P	N/P
CK	12.7c	87.8b	6.9b
N	11.7d	94.2a	8.0a
NP	12.7c	72.0c	5.7c
NPK	13.2bc	74.2c	5.6c
NPKM1	14.8a	78.1c	5.3c
NPKM2	13.6b	76.6c	5.6c

Note: Different letters in the same column indicate significant differences among fertilizer treatments at the *p <* 0.05 level.

### Soil MBC, SBR and cumulative mineralized C

**Fig 1:**
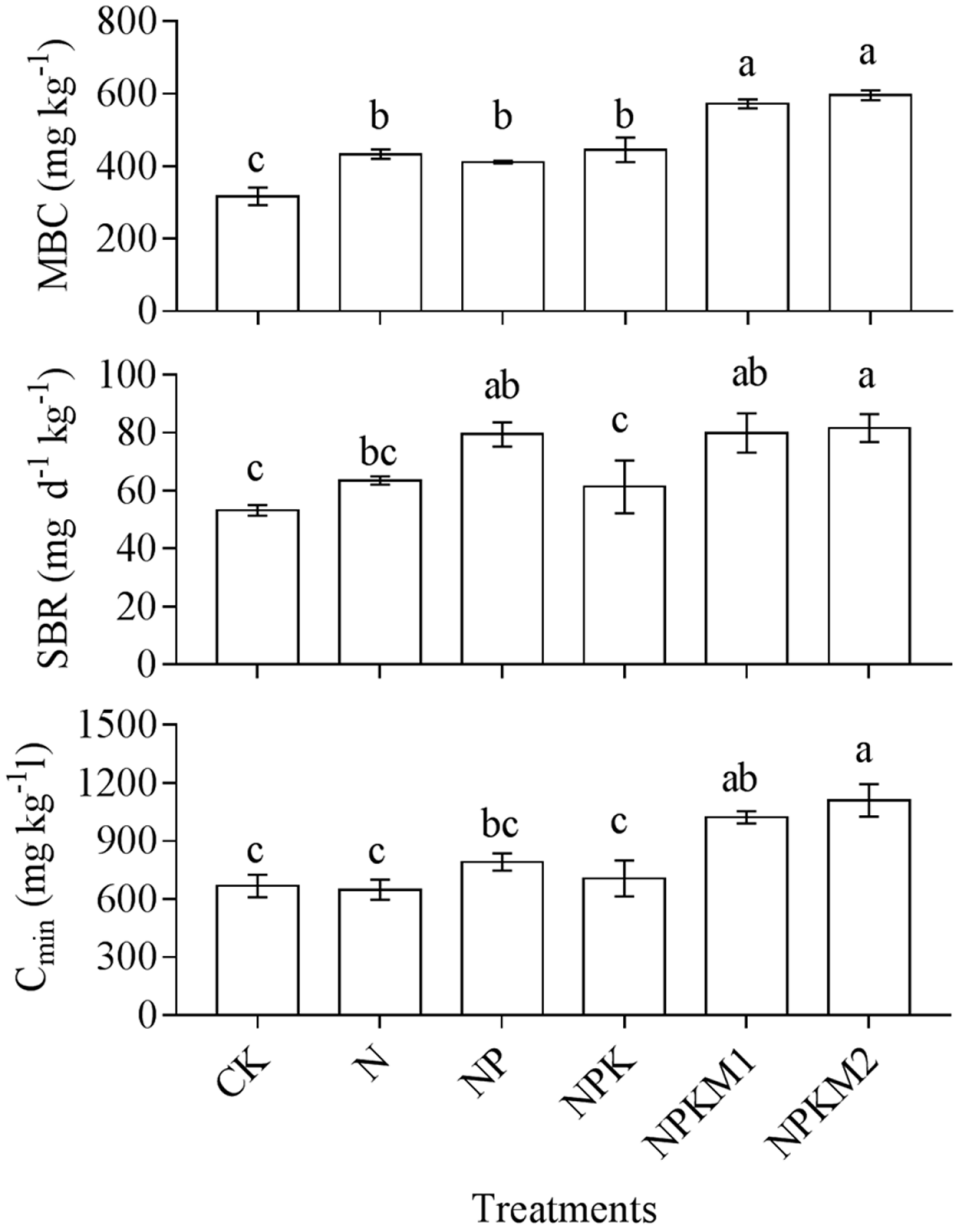
Soil MBC, SBR and C_min_ under different fertilization treatments. Vertical bars represent the SE (n = 3) and lowercase letters indicate significant differences among fertilizer treatments at the *p <* 0.05 level.

Soil MBC, SBR and cumulative mineralized C (C_min_) are presented in Fig 1. The MBC of the integrated fertilization treatments (NPKM1 and NPKM2) was significantly higher than that of the other treatments, and the MBC of the CK treatment was significantly lower than that of the others. The SBR of NPKM1, NPKM2 and NP was significantly higher than that of CK and NPK. The C_min_ of the integrated fertilization treatments (NPKM1 and NPKM2) was significantly higher than that of the CK, N and NPK treatments.

### Soil enzymes activities

**Fig 2:**
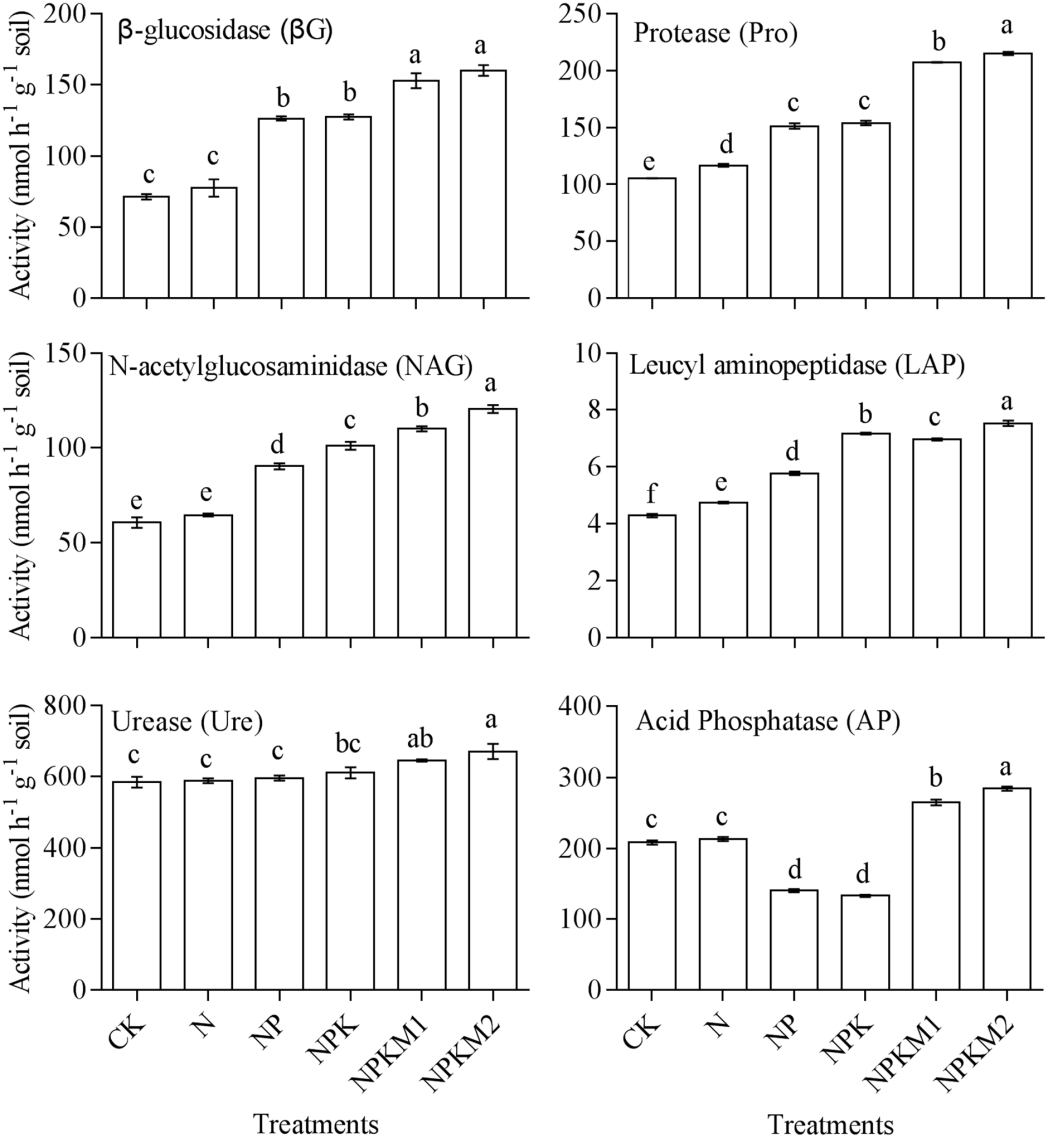
Soil enzyme activities of different fertilization treatments. Vertical bars represent the SE (n = 3) and lowercase letters indicate significant differences among fertilizer treatments at the *p <* 0.05 level.

The soil enzyme activities of the different fertilization treatments are shown in Fig 2. The activities of βG, protease, NAG, LAP, urease and AP of the NPKM1 and NPKM2 treatments were significantly higher than those of the CK, N and NP treatments. The enzymes activities of NPK were significantly lower than those of NPKM2. The protease, NAG and AP activities of NPKM1 were significantly higher than those of NPK. The protease, NAG, LAP and AP activities of NPKM1 were significantly lower than those of NPKM2. The AP activities of the NP and NPK treatments were significantly lower than those of the other treatments. Long-term integrated fertilization with organic manure and chemical fertilizers increased soil enzyme activities.

### Soil enzymatic stoichiometric characteristics

The soil enzymatic stoichiometric characteristics of different treatments are presented in Table 4. The values of the soil lnβG/ln(NAG+LAP), lnβG/lnUre and lnβG/lnPro under CK and N treatments were relatively lower, and there was not a significant difference between the rest of the treatments. The soil lnPro/ln(NAG+LAP) under NPK was significantly lower than that of the other treatments, followed by NP which significantly lower than that of the N and NPKM1 treatments. The soil lnPro/lnUre under treatments with organic fertilizers (NPKM1 and NPKM2) was significantly higher than that of the other treatments. The soil ln(NAG+LAP)/lnAP and lnPro/lnAP under the NP and NPK treatments were significantly higher than those of the other treatments. The soil ln(NAG+LAP)/lnAP and lnPro/lnAP under integrated fertilization with organic manure and chemical fertilizers were significantly higher than those of CK and N treatments, while the values of lnUre/lnAP showed the opposite trend. The soil lnβG/lnAP under the NP and NPK treatments was significantly higher than that of the other treatments.

**Table 4:** Soil enzymatic stoichiometric characteristics of different treatments.

Treatments	lnβG/ln(NAG+LAP)	lnβG/lnUre	lnβG/lnPro	lnβG/lnAP	lnPro/ln(NAG+LAP)	lnPro/lnUre	ln(NAG+LAP)/lnAP	lnPro/lnAP	lnUre/lnAP
CK	1.023b	0.670b	0.916b	0.799c	1.117ab	0.731d	0.781d	0.873e	1.193b
N	1.025b	0.681b	0.912b	0.810c	1.123a	0.747c	0.791d	0.888d	1.189b
NP	1.060a	0.757a	0.964a	0.979a	1.099b	0.785b	0.924b	1.015b	1.293a
NPK	1.035ab	0.756a	0.962a	0.991a	1.075c	0.785b	0.958a	1.030a	1.312a
NPKM1	1.056ab	0.777a	0.943ab	0.901b	1.120a	0.824a	0.854c	0.956c	1.160c
NPKM2	1.046ab	0.780a	0.945a	0.898b	1.107ab	0.825a	0.859c	0.951c	1.152c

Note: In the table below, lnβG/ln(NAG+LAP), lnβG/lnUre and lnβG/lnPro refer to the acquisition of C related to N; lnβG/lnAP refers to the acquisition of C related to P; ln(NAG+LAP)/lnAP, lnPro/lnAP and lnUre/lnAP refer to the acquisition of N related to P; lnPro/ln(NAG+LAP) and lnPro/lnUre refer to the N acquisition from different sources. Different letters in the same column indicate significant differences among fertilizer treatments at the *p <* 0.05 level.

### Correlations between soil enzymatic, microbial and chemical properties

Correlation coefficients between the soil enzymatic, microbial and chemical properties are presented in Table 5. Soil βG, protease, NAG and LAP activities were significantly (*P* < 0.05, *P* < 0.01) and positively related to soil TP and SOC. Soil urease activity and MBC were significantly (*P* < 0.01) and positively related to SOC. Soil SBR and cumulative C_min_ were significantly (*P* < 0.05, *P* < 0.01) and positively related to TP and SOC. Soil Ah-N concentration was significantly (*P* < 0.01 or *P* < 0.05) and positively related to the activities of soil βG, protease, NAG and urease, MBC, SBR and C_min_. Soil Olsen-P concentration was significantly (*P* < 0.01 or *P* < 0.05) and positively related to the activities of soil βG, protease, NAG, LAP and urease, MBC, SBR and C_min_.

**Table 5:** Pearson correlation coefficients between soil enzymes, MBC, SBR, C_min_ and TN, TP, SOC, Ah-N, Olsen-P, pH.

Coefficient	TN	TP	SOC	Ah-N	Olsen-P	pH
βG	0.545	0.985**	0.940**	0.826*	0.985**	-0.344
Pro	0.583	0.918**	0.986**	0.878*	0.963**	-0.249
NAG	0.541	0.961**	0.926**	0.813*	0.965**	-0.356
LAP	0.490	0.904*	0.852*	0.768	0.895*	-0.446
Ure	0.592	0.808	0.947**	0.828*	0.898*	-0.140
AP	0.385	0.202	0.582	0.520	0.378	0.286
MBC	0.715	0.781	0.969**	0.960**	0.846*	-0.394
SBR	0.736	0.856*	0.886*	0.863*	0.867*	-0.385
C_min_	0.580	0.830*	0.954**	0.813*	0.917**	-0.042

Note:

** and * in the table represent significant differences at the *p* < 0.01 or *p* < 0.05 level, respectively.

### Correlation matrix with soil microbial and biochemical parameters

Correlation coefficients between soil microbial and biochemical parameters are shown in Table 6. The soil enzyme (except for AP) activities were significantly (*P* < 0.05 or *P* < 0.01) and positively related to each other. In addition, the soil MBC was significantly (*P* < 0.05 or *P* < 0.01) and positively related to soil enzyme activities (except for AP). The SBR was significantly (*P* < 0.05) and positively related to the protease and βG activities. The cumulative C_min_ was significantly (*P* < 0.05 or *P* < 0.01) and positively related to the soil βG, protease, NAG, urease activities, MBC and SBR.

**Table 6:** Correlation matrix with soil microbial and biochemical parameters.

Coefficient	βG	Protease	NAG	LAP	Urease	AP	MBC	SBR	C_min_
βG	1	0.967**	0.988**	0.940**	0.881*	0.320	0.863*	0.839*	0.878*
Protease		1	0.959**	0.894*	0.959**	0.548	0.943**	0.838*	0.957**
NAG			1	0.976**	0.905*	0.326	0.871*	0.766	0.860*
LAP				1	0.847*	0.219	0.830*	0.641	0.747
Urease					1	0.688	0.936**	0.716	0.958**
AP						1	0.643	0.381	0.709
MBC							1	0.787	0.894*
SBR								1	0.831*
C_min_									1

Note:

** and * in the table represent significant differences at the *p* < 0.01 or *p* < 0.05 level, respectively.

## Discussion

Results showed that contrasting fertilization treatments since 1983 had induced considerable differences in soil fertility. After 30-years of fertilizer application, the SOC of the integrated fertilization treatments was significantly higher than that of the no organic manure treatments. This result is in agreement with other long-term experiments which have found that the application of organic manure can promote the accumulation of SOM and the major soil macronutrients N, P, and K and increase both the soil’s microbial biomass and activity [2,41]. At the same nutrient dose, the application of integrated fertilization with organic manure and chemical fertilizer increased TN, TP, TK, SOC, Ah-N, Olsen-P and EK and played a more prominent role in increasing soil nutrient pools, improving soil fertility [42] and reducing nitrogen and phosphorus losses [43] relative to chemical fertilization alone. The literature has reported that soil pH decreases after the long-term application of chemical nitrogen fertilizers [44,45]. Ai et al. reported that soil pH values were not influenced by long-term (31-year) fertilizer treatments [2]. In this experiment, the application of chemical fertilizers alone decreased soil pH, but the differences between treatments did not reach significant levels.

In this experiment, enzyme activities showed similar increasing tendencies in response to long-term organic manure fertilization despite the environmental complexity of soil and the heterogeneity of the enzymes. Integrated fertilization with organic and chemical fertilizers increased soil enzyme activities. Protease, NAG and βG activities were significantly increased by the application of organic manure. There was not significant difference in urease activity between treatments without organic manure. Although N fertilization may alleviate soil N-limitation, a single inorganic N fertilization may disturb the balance of inorganic N and organic N and suppress the increases in soil enzyme activities and microbial biomass in the end [46]. Acid phosphatase is very important in soil phosphorus metabolism, coming mainly from root secretions and plant residues. The increase in phosphomonoesterase activities in soil might be related to the stimulation of microbial growth and/or to the decrease in the concentration of available P [47]. In this experiment, the addition of chemical phosphorus fertilizers increased the soil Olsen-P and TP and suppressed AP activity, while the addition of organic manure increased AP activity and Olsen-P significantly. The results indicate that the addition of organic manure can increase soil Olsen-P while maintaining AP activity.

Long-term integrated fertilization with organic manure and chemical fertilizers affected the biomass and function of soil microorganisms, too. As an important component of the C pool, soil microbial biomass carbon plays a key role in the C cycle in soil [48,49]. The application of organic fertilizer increased the MBC, enhanced the SBR and accelerated the mineralization of soil organic carbon [6,50]. At the same nutrient doses applied in this experiment, integrated fertilization with organic manure and chemical fertilizers significantly increased MBC, SBR and cumulative C_min_ more than chemical fertilizers alone.

As shown in Tables 3 and 4, the soil C/N ratios of the treatments with organic manure (NPKM1, NPKM2) were significantly higher than those of treatments CK, N and NP. The soil C/N, lnβG/ln(NAG+LAP) and lnβG/lnUre of NPKM1 were higher than those of NPK. These results suggest that the application of organic manure increased C acquisition and improved the utilization of soil organic matter by microorganisms. The soil lnβG/lnPro ratios of NPKM1 and NPKM2 were lower than those of the NPK treatment, while the lnPro/ln(NAG+LAP) ratios were higher, indicating that microorganisms showed selectivity in N substrates. The oil N/P ratios under N and CK treatments were significantly higher than those of the other treatments. The soil ln(NAG+LAP)/lnAP ratios of treatments with organic manure were significantly lower than those of the NPK and NP treatments but were higher than those of CK and N. The lnPro/lnAP ratios of NPKM1 and NPKM2 were lower than those of NPK and NP. The soil lnUre/lnAP ratios under NPKM1 and NPKM2 were significantly lower than those under the other treatments. These results suggest that the regulatory relationships between nutrient supply and nutrient mineralization are asymmetric for N and P [17] and that both organic fertilization and chemical P fertilization may cause a greater enrichment of P in soil than enrichment of N. The soil C/P ratio under N treatment was significantly higher than that in the other treatments. The acquisition of C relative to organic P is indicated by ratio of lnβG/lnAP. The ratios of lnβG/lnAP under the NP and NPK treatments were significantly higher than those of the other treatments, while the ratios of the integrated fertilization treatments (NPKM1 and NPKM2) were significantly higher than those of the CK and N treatments. This finding suggested that long term single chemical N fertilization or no fertilization may result in a soil P deficiency. Long-term chemical P fertilizer applications could result in P accumulation in the soil and an intensified microbial demand for C, and integrated fertilization with organic manure and chemical fertilizers increased P acquisition greater relative to C acquisition. Meanwhile, the lnβG/lnUre and lnβG/lnPro ratios of the treatments with P fertilizers applied (NP, NPK, NPKM1, NPKM2) were significantly higher than those of CK and N (except for the insignificant differences between NPKM1, NP and NPK for lnβG/lnPro), suggesting that P fertilization increased the microbial C acquisition [23]. For many heterotrophic organisms, a high growth rate not only corresponds to low C/N and C/P ratios but also corresponds to a low N/P ratio [51–53]. In this study, chemical P fertilizer applications decreased C/N and C/P ratios, which were inversely proportional with the organic matter decomposition, meaning that chemical P applications accelerated the mineralization of organic matter. In addition, low N/P ratios exacerbated microbial N limitation, regulating the function of enzymes to obtain N in order to meet the growth demands of microorganisms. Thus, the ln(NAG+LAP)/lnAP, lnPro/lnAP and lnUre/lnAP ratios of the NP and NPK treatments were significantly higher than those of the other treatments, promoting soil nitrogen fixation to improve the soil N pool.

The activities of βG, NAG, LAP and AP show similarly scaling relationships when catalysing the hydrolysis of assimilable products from environmental sources of C, N and P, with a mean ratio for C:N:P activities near 1:1:1 in all habitats [24]. In this study, the stoichiometric ratios of lnβG/ln(NAG+LAP)/lnPro/lnUre/lnAP ranged from 1:0.94:1.04:0.67:1.01 to 1:0.98:1.10:0.78:1.25. It indicated that the acquisition of C, N and P changed consistently and synchronously under different nutrient management regimes. The stoichiometric ratios of lnβG/lnUre and lnPro/lnUre ranged from 0.67 to 0.78 and 0.73 to 0.83 under different nutrient management systems, respectively. Although part of urease substrate was from the chemical urea fertilizers rather than protein hydrolysates or humic substances, the activity of urease still showed similar and relatively synchronous variations with the activities of protease and βG under different nutrient management regimes. Considering the above results, it is reasonable and necessary to take protease and urease into consideration if we want to thoroughly study nutrient cycles and material transformation in soil, especially for the eco-enzymatic stoichiometric assessment of the hydrolysis of proteins, low-molecular organic compounds such as amino acids, carbohydrates and phosphomonoesters in soil. Protease hydrolyses protein and provides substrates for NAG and LAP. Variations in protease activity provide a theoretical basis for the explanation the changes in NAG and LAP activities. Soil microorganism biomass and microbial activity was significantly and positively related to protease, and there is a mutual promotional relationship between them.

Soil enzyme activity represents an intersection of the ecological stoichiometry theory and the metabolic theory of ecology [24], wherein enzyme activity links environmental nutrient availability with microbial production. Soil TP and SOC provide substrates for enzymes and nutrients for microorganisms, therefore soil enzyme activities and MBC were significantly positively related to them. The increases in enzyme activity, microbial biomass and SBR improved soil nutrients, and the concentrations of soil Ah-N and Olsen-P were significantly and positively related to the activities of soil βG, protease, NAG and urease, MBC, SBR and C_min_. In this study, SBR was only significantly and positively correlated to protease and βG activities, indicating that protease and βG can hydrolyse proteins and chitin into available C sources for microorganisms and increase C_min_ as well. Soil NAG plays a role in the degradation of chitin. Proteins, chitin and peptidoglycan are the principal reservoirs of organic N [54]. The hydrolysis process is accompanied by the release of C resources, hence cumulative C_min_ was significantly and positively correlated to soil urease, protease, βG and NAG activities. Microorganisms are the basis of carbon mineralization, while MBC and SBR reflect the microbial biomass and activity. Therefore, the cumulative C_min_ was significantly and positively correlated to soil MBC and SBR.

In summary, our results suggest that integrated fertilization with organic manure and chemical fertilizers can increase soil enzyme activities and improve the biomass and activity of soil microorganisms, resulting in improvements to soil fertility and nutrient supply. The variations of eco-enzymatic stoichiometric characteristics in paddy soil as affected by long-term different nutrient managements suggest that soil protease and urease activities also showed similar and relatively synchronous variation with the activities of soil βG, NAG, LAP and AP activities, and should be taken into consideration in further eco-enzymatic stoichiometric assessments of organic nutrient compounds. Integrated fertilization with chemical fertilizers and organic manure should be recommended as a preferable nutrient management solution for intensive and sustainable rice production.

## Supporting information

S1 Table Soil nutrient concentrations and pH values after long-term different fertilization (mean ± SD). (DOCX)

S2 Table Soil MBC, SBR and C_min_ under different fertilization treatments (mean ± SD). (DOCX)

S3 Table Soil enzyme activities of different fertilization treatments (mean ± SD). (DOCX)
