# Supplementary material for: Variations in eco-enzymatic stoichiometric and microbial characteristics in paddy soil as affected by long-term integrated organic-inorganic fertilization
Source: PLoS One. 2017 Dec 18;12(12):e0189908. doi: 10.1371/journal.pone.0189908 (PMC5734689; doi:10.1371/journal.pone.0189908)
Supplement: S1 Table — (DOCX) [file pone.0189908.s001.docx]

**S1 Table. Soil nutrient concentrations and pH values after long-term different fertilization (mean ± SD)**

| Treatments | Total nutrient (g kg^-1^) | | | Available nutrient (mg kg^-1^) | | | SOC | pH |
| --- | --- | --- | --- | --- | --- | --- | --- | --- |
|  | TN | TP | TK | Ah-N | Olsen-P | EK | (g kg^-1^) |  |
| CK | 1.34±0.02 | 0.43±0.01 | 14.5±0.1 | 191.6±3.5 | 10.1±0.7 | 31.6±1.2 | 14.6±0.4 | 6.10±0.07 |
| N | 1.58±0.03 | 0.44±0.00 | 14.6±0.1 | 237.3±2.2 | 9.9±0.7 | 30.9±2.0 | 15.9±0.2 | 6.02±0.03 |
| NP | 1.55±0.04 | 0.61±0.02 | 13.8±0.3 | 235.1±1.3 | 24.6±0.7 | 29.8±1.2 | 16.9±0.4 | 6.03±0.15 |
| NPK | 1.46±0.04 | 0.58±0.03 | 15.0±0.4 | 230.2±2.7 | 21.6±1.7 | 40.1±1.0 | 16.6±0.2 | 6.02±0.02 |
| NPKM1 | 1.50±0.05 | 0.63±0.01 | 15.3±0.8 | 260.9±3.7 | 29.4±1.0 | 42.8±1.1 | 19.0±0.3 | 6.05±0.15 |
| NPKM2 | 1.68±0.05 | 0.66±0.02 | 15.7±0.6 | 267.5±2.3 | 35..1±1.8 | 43.9±2.1 | 19.5±0.2 | 6.04±0.03 |
