# Supplementary material for: Variations in eco-enzymatic stoichiometric and microbial characteristics in paddy soil as affected by long-term integrated organic-inorganic fertilization
Source: PLoS One. 2017 Dec 18;12(12):e0189908. doi: 10.1371/journal.pone.0189908 (PMC5734689; doi:10.1371/journal.pone.0189908)
Supplement: S2 Table — (DOCX) [file pone.0189908.s002.docx]

**S2 Table. Soil MBC, SBR and C_min_ under different fertilization treatments** **(mean ± SD)**

| Treatments | MBC (mg kg^-1^) | SBR (mg CO_2_-C d^-1^ kg^-1^) | C_min_ (mg CO_2_-C kg^-1^) |
| --- | --- | --- | --- |
| CK | 317.3±42.2 | 53.2±3.1 | 667.4±102.4 |
| N | 433.7±22.2 | 63.5±2.4 | 647.9±90.6 |
| NP | 412.3±5.9 | 79.4±7.3 | 790.6±76.6 |
| NPK | 445.8±58.5 | 61.3±15.8 | 707.4±161.6 |
| NPKM1 | 572.3±21.0 | 79.9±11.8 | 1022.3±55.4 |
| NPKM2 | 596.2±22.8 | 81.6±8.3 | 1109.6±144.7 |
