# Supplementary material for: Variations in eco-enzymatic stoichiometric and microbial characteristics in paddy soil as affected by long-term integrated organic-inorganic fertilization
Source: PLoS One. 2017 Dec 18;12(12):e0189908. doi: 10.1371/journal.pone.0189908 (PMC5734689; doi:10.1371/journal.pone.0189908)
Supplement: S3 Table — (DOCX) [file pone.0189908.s003.docx]

**S3 Table. Soil enzyme activities of different fertilization treatments (mean ± SD)**

| Treatments | Enzyme activities (nmol g^-1^ h^-1^, mean ± SD) | | | | | |
| --- | --- | --- | --- | --- | --- | --- |
|  | βG | Pro | NAG | LAP | Ure | AP |
| CK | 71.4±3.2 | 105.5±0.3 | 60.7±4.8 | 4.29±0.10 | 584.6±26.3 | 208.3±5.3 |
| N | 77.5±10.6 | 116.9±2.3 | 64.6±1.3 | 4.75±0.06 | 588.3±11.9 | 213.2±5.0 |
| NP | 126.4±2.4 | 151.3±4.1 | 90.4±2.7 | 5.78±0.10 | 596.2±12.6 | 140.3±3.6 |
| NPK | 127.4±3.0 | 154.0±3.4 | 101.2±3.5 | 7.17±0.06 | 610.9±27.3 | 133.0±2.9 |
| NPKM1 | 152.9±9.0 | 207.4±0.5 | 110.1±2.2 | 6.97±0.06 | 645.6±5.3 | 264.7±7.2 |
| NPKM2 | 160.2±6.5 | 215.2±2.5 | 120.5±3.6 | 7.53±0.16 | 670.7±36.9 | 284.2±5.1 |
